# Central Dental Association of Northern New Jersey

**Published:** 1893-02

**Authors:** S. C. G. Watkins


### CENTRAL DENTAL ASSOCIATION OF NORTHERN NEW JERSEY.

TO THE EDITOR OF THE INTERNATIONAL DENTAL JOURNAL:

SIR,—At a regular bi-monthly meeting of the Central Dental Association of Northern New Jersey, held in Newark, N. J., December 19, 1892, the proceedings were made unusually interesting by the presentation of a handsome gold watch and chain to the Secretary, Dr. Charles A. Meeker. The following remarks were made by Dr. S. C. G. Watkins in presenting the gift:

"MR. PRESIDENT, MEMBERS, AND FRIENDS OF THE CENTRAL DENTAL ASSOCIATION OF NORTHERN NEW JERSEY,—There devolves upon your chairman of the Executive Committee this evening a duty more pleasant than he is often called upon to perform,—that of expressing, as well as he is able, the feeling which the members of this society entertain towards those who have aided them.

"It is not necessary to dwell at this time, however we may be tempted to do so, upon our past history. For something like ten years we have maintained a series of meetings more regular, better attended, and more profitable than has usually been the fate of similar societies. I do not mention this to boast, nor to make invidious distinctions, nor to impress upon any of our good friends, not members of this Association, that they should feel humble and reverent in our presence,—far from it. I mention the fact because it throws lustre upon a certain name which every member of this Association delights to honor. Such success as we have had has not come by chance, nor because our members are more able, more devoted to their profession, more capable of imparting to others the benefits of their experience, than are dentists in other parts of the country. It is rather because of the skill which has planned our meetings and contrived our entertainments so as to bring here those who could instruct us, and attract those best capable of profiting by such instruction. I shall not ask who has done this, as it might bring a blush upon the cheek of one of our number,—than whom the society does not contain a more modest or a *meeker* man.

"You all doubtless know, moreover, how anxious we have all been to express, in some fitting way, our gratitude to the man who has done so much for us, and how that desire was made possible by our friend Dr. Stockton, who conceived the idea and gave birth to the expression, so that it took practical shape in the purchase of a slight testimonial, to be given him as a tangible evidence of our gratitude.

"This article was selected not by any means because it was deemed necessary to put a watch on the treasurer,—far from that,—but rather that you, sir, may be constantly reminded of this society as you note the flight of time, and by the union of the two thoughts thus made necessary, may never forget the approach of meeting-nights, nor neglect in the future those genial offices you have performed so faithfully and so pleasingly in the past. Please accept this token of appreciation from the members of the Central Dental Association of Northern New Jersey, and may every revolution of its hands around the dial find you well, happy, and prosperous for many years to come."

Dr. Meeker responded as follows:

"Gentlemen and Friends of the Central Dental Association,—This is a most unlooked-for evidence of your esteem. You do not know how I appreciate it. I shall think of the Association much oftener than the opportunities afforded by an occasional glance at this beautiful watch. I want to say that our next meeting in February will be the grandest and most unique meeting ever held in this country. Then many of the most learned and eminent men in the profession will be present to reply to the toasts of the various States. I need not add that this digression has not made me forget the splendid recognition you have just shown me, and I thank you for it most cordially."

Engraved on the inside of the watch-case is this inscription:

"Presented to Dr. C. A. Meeker by the Central Dental Association of Northern New Jersey, December 19, 1892, as a testimonial of faithful services rendered as treasurer."

On the outside of the case is the doctor's monogram.

After this the meeting was called to order and the regular business of the evening was taken up.

Yours truly,
S. C. G. Watkins.

